# Evolution, Interspecies Transmission, and Zoonotic Significance of Animal Coronaviruses

**DOI:** 10.3389/fvets.2021.719834

**Published:** 2021-10-18

**Authors:** Prapti Parkhe, Subhash Verma

**Affiliations:** Department of Veterinary Microbiology, DGCN College of Veterinary and Animal Sciences, Chaudhary Sarwan Kumar Himachal Pradesh Krishi Vishvavidyalaya, Palampur, India

**Keywords:** coronavirus, evolution, interspecies transmission, host cell receptor, protein binding motif, zoonotic animal origin, classification, animal coronaviruses

## Abstract

Coronaviruses are single-stranded RNA viruses that affect humans and a wide variety of animal species, including livestock, wild animals, birds, and pets. These viruses have an affinity for different tissues, such as those of the respiratory and gastrointestinal tract of most mammals and birds and the hepatic and nervous tissues of rodents and porcine. As coronaviruses target different host cell receptors and show divergence in the sequences and motifs of their structural and accessory proteins, they are classified into groups, which may explain the evolutionary relationship between them. The interspecies transmission, zoonotic potential, and ability to mutate at a higher rate and emerge into variants of concern highlight their importance in the medical and veterinary fields. The contribution of various factors that result in their evolution will provide better insight and may help to understand the complexity of coronaviruses in the face of pandemics. In this review, important aspects of coronaviruses infecting livestock, birds, and pets, in particular, their structure and genome organization having a bearing on evolutionary and zoonotic outcomes, have been discussed.

## Introduction

Coronaviruses (CoVs) form the subfamily *Orthocoronavirinae* of family *Coronaviridae* under order *Nidovirales* and realm *Riboviria*. These are pleomorphic, enveloped, single molecule of linear, positive-sense, single-stranded RNA viruses containing genome sizes of around 30 kb among known RNA viruses (1). The club-shaped peplomers composed of large viral glycoprotein (spike or S protein responsible for attachment to cells) projecting from the envelope give a crown-like appearance of the virus under a transmission electron microscope, thus named corona meaning crown. CoV was considered a minor pathogen of the respiratory tract until 2002 in humans (2). The increased interest in its replication, transmission, pathogenesis, and distribution was pursued after an outbreak linked to the emergence of a new CoV [severe acute respiratory syndrome (SARS)-CoV] causing SARS after 2002 (2–5). Another virus called Middle East respiratory syndrome CoV (MERS-CoV) in 2014, distinct from SARS-CoV, was isolated from an outbreak of severe respiratory infection in the Middle East (6). On the other hand, an acute respiratory infection caused by an avian CoV, later named as infectious bronchitis virus with high mortality (40–90%), had shown up in the late 1920s and was the earliest report of a CoV infection in animals (7, 8). Currently, the SARS-CoV-2 and its mutated strains predominantly infecting humans with contentious animal origin have created a platform for researchers to study its genomics in-depth. Animal species play an important role as a host or reservoir in the transmission cycle of CoV, and specific receptors on their cell provide essential factors for replication and mutations within the genome of CoVs. CoVs infect various animal species ranging from livestock, poultry, cats, dogs, mice, bats, pangolins, wild felids, and other species of animals such as minks, rabbits, ducks, guinea fowls, gooses, beluga whales, etc., (9–12). These mammals are frequently studied for understanding their coevolution with human CoVs, interspecies transmission, and the emergence of new mutant strains. CoV infection in animals is mainly associated with respiratory and gastrointestinal systems resulting in mild to fatal diseases. The bovine, avian, and porcine animal groups form a major part of production industries, and in like manner, the canine and feline species have paramount importance as pets commercially due to their high demand in society. The incidence of diseases in these animal species represents a threat to the animal welfare, environment, public health, and economy, reflecting as losses in productivity, trade, market value, control costs, and food security. In this review, CoVs infecting important livestock, poultry, and pets have been discussed in relation to their structure and genome organization having a bearing on evolutionary and zoonotic outcomes.

## Coronavirus Structure, Major Proteins, and Their Functions

**Figure 1:**
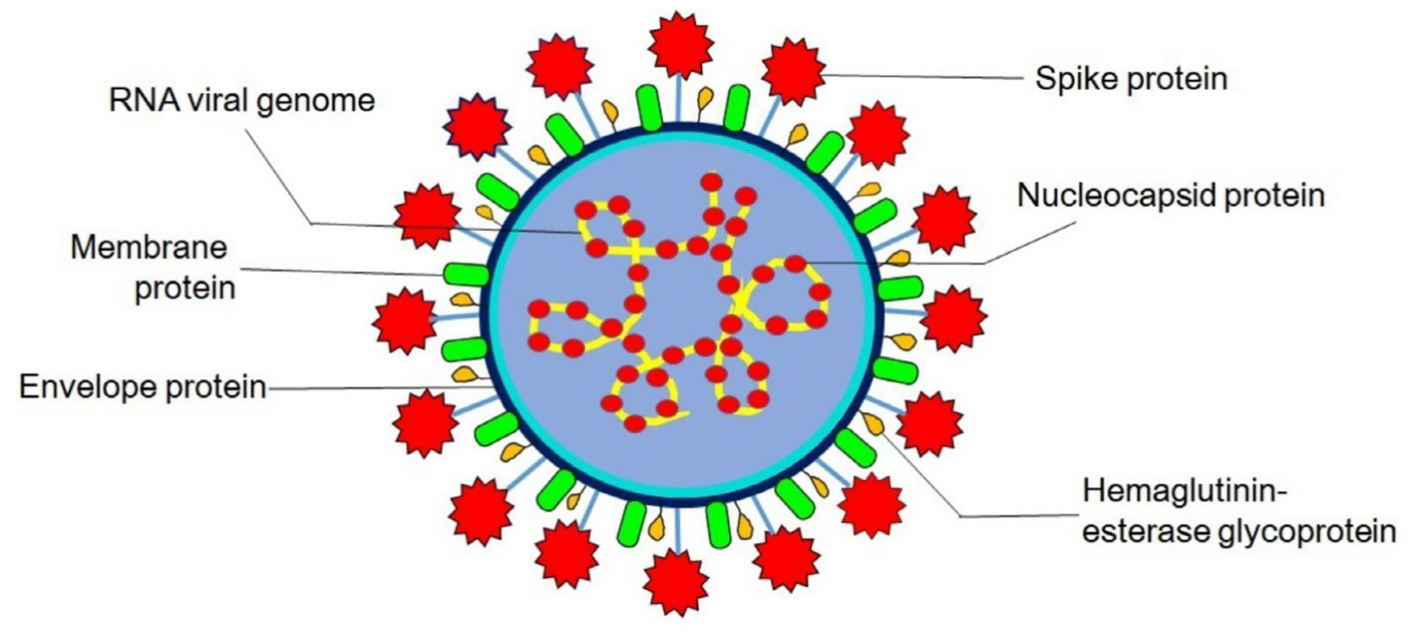
Structure of coronavirus. Hemagglutinin-esterase glycoprotein is exclusively present in members of Betacoronavirus (human and bovine coronavirus).

Virions are roughly spherical and enveloped with marked spike (S) proteins that identify various specific host cell receptors and co-receptors for attachment, fusion, and entry of the virus into the cell. In addition to S proteins, other structural proteins are nucleocapsid (N) proteins, the most abundant membrane (M) proteins, envelope (E) proteins, and other non-glycosylated envelope proteins present in lower quantities, which help in the formation of an envelope. The flexible nucleocapsid within the envelope consists of genomic RNA linked to the nucleoprotein (Figure 1).

The functions of these major structural proteins of CoV are stated in Table 1.

**Table 1:** Major proteins of coronavirus and their functions.

**Major protein**	**Function**
Nucleocapsid protein (N)	Multifunctional protein
	Forms complexes with genome RNA to make up nucleocapsid
	Interacts with the membrane proteins during virus life cycle, especially for virion assembly and viral budding
	Enhances the efficiency of virus transcription
	Principle target for vaccine development as a major immunogen (13)
	An important diagnostic marker for coronavirus disease (13, 14)
Spike or peplomer protein (S)	Critical for binding to host cell receptors to facilitate entry into host cell (15)
Envelope protein (E)	Smallest of the major proteins
	Interacts with M protein to form viral envelop
	Expressed abundantly inside the infected cell but only a small portion incorporated into virion envelope (16)
	A majority is localized at the site of intracellular trafficking, i.e., the ER, Golgi, and ER–Golgi intermediate compartment where it participates in virus assembly and budding (17)
Membrane protein (M)	Most abundant structural protein
	Central organizer of CoV assembly (18, 19)
	Defines shape of viral envelope
	Responsible for transmembrane transport of nutrients and bud release
Hemagglutinin-esterase glycoprotein	Mediate reversible attachment to sialic acids
	Act both as carbohydrate-binding lectin and as a receptor-destroying enzyme (RDE) (20)
	Responsible for enzymatic activity
	Lack membrane fusion activity
	Accessory to spike glycoprotein in virion (21)

## Classification of Coronavirus

Initially, the classification was based on their serological and antigenic properties—groups 1, 2, and 3 as opposed to newly revised taxonomy based on the level of viral genetic phylogeny. The phylogenetic analysis for classification of CoV is usually acquired by using short fragments of several conserved genes that are present in all CoV genomes and are of a significant length, such as Pol (RNA-dependent RNA polymerase), N (nucleoprotein), S (spike protein), and chymotrypsin-like protease and helicase. The envelope and membrane genes are not used in phylogenetic studies due to their short lengths (1, 22). Furthermore, complete genome sequence and proteomic approaches are also carried out to construct the phylogenetic tree of CoVs. Now, the subfamily *Orthocoronavirinae* is classified into four genera: alpha, beta, gamma, and delta CoVs infecting a wide variety of animal and avian species (23, 24). *Betacoronavirus* genus is further classified into lineages A, B, C, and D (1) and other subgenus *Hibecovirus* (25). The list of important CoV species classified under individual genera is given later (Table 2). Apart from this, several other animal species that harbor the CoVs are rodents, rabbits, bats, pangolin, ferrets, mink, snake, frogs, marmots, hedgehogs, and many other wild animals, as carriers or reservoirs that may need attention regarding zoonotic interventions (26–33).

**Table 2:** Important coronavirus species within individual genera.

**Genus**	**Species**
**ALPHACORONAVIRUS**	
	Human coronavirus 229E
	Human coronavirus NL63
	Porcine epidemic diarrhea virus (PEDV)
	Transmissible gastroenteritis virus (TGEV)
	Porcine respiratory coronavirus (PRCV)
	Feline infectious peritonitis virus (FIPV)
	Canine enteric coronavirus (CCoV)
	Rhinolophus bat coronavirus HKU2
**BETACORONAVIRUS SUBGENUS**	
Embecovirus (Lineage A)	Human coronavirus HKU1
	Human coronavirus OC43
	Bovine coronavirus (BCoV)
	Porcine hemagglutinating encephalomyelitis virus (PHEV)
	Canine respiratory coronavirus (CRCoV)
	Feline enteric coronavirus (FCoV)
	Murine hepatitis virus (MHV)
Sarbecovirus (Lineage B)	Severe acute respiratory syndrome (SARS) related coronavirus (SARS-CoV-1, SARS-CoV-2)
Merbecovirus (Lineage C)	Middle east respiratory syndrome (MERS) related coronavirus
Nobecovirus (Lineage D)	Bat coronaviruses
Hibecovirus	Bat Hp-betacoronavirus Zhejiang 2013
**GAMMACORONAVIRUS**	
	Infectious bronchitis virus (IBV)
	Bluecomb virus of turkey
**DELTACORONAVIRUS**	
	Porcine deltacoronavirus (PdCV)
	Avian coronaviruses

## Life Cycle

**Figure 2:**
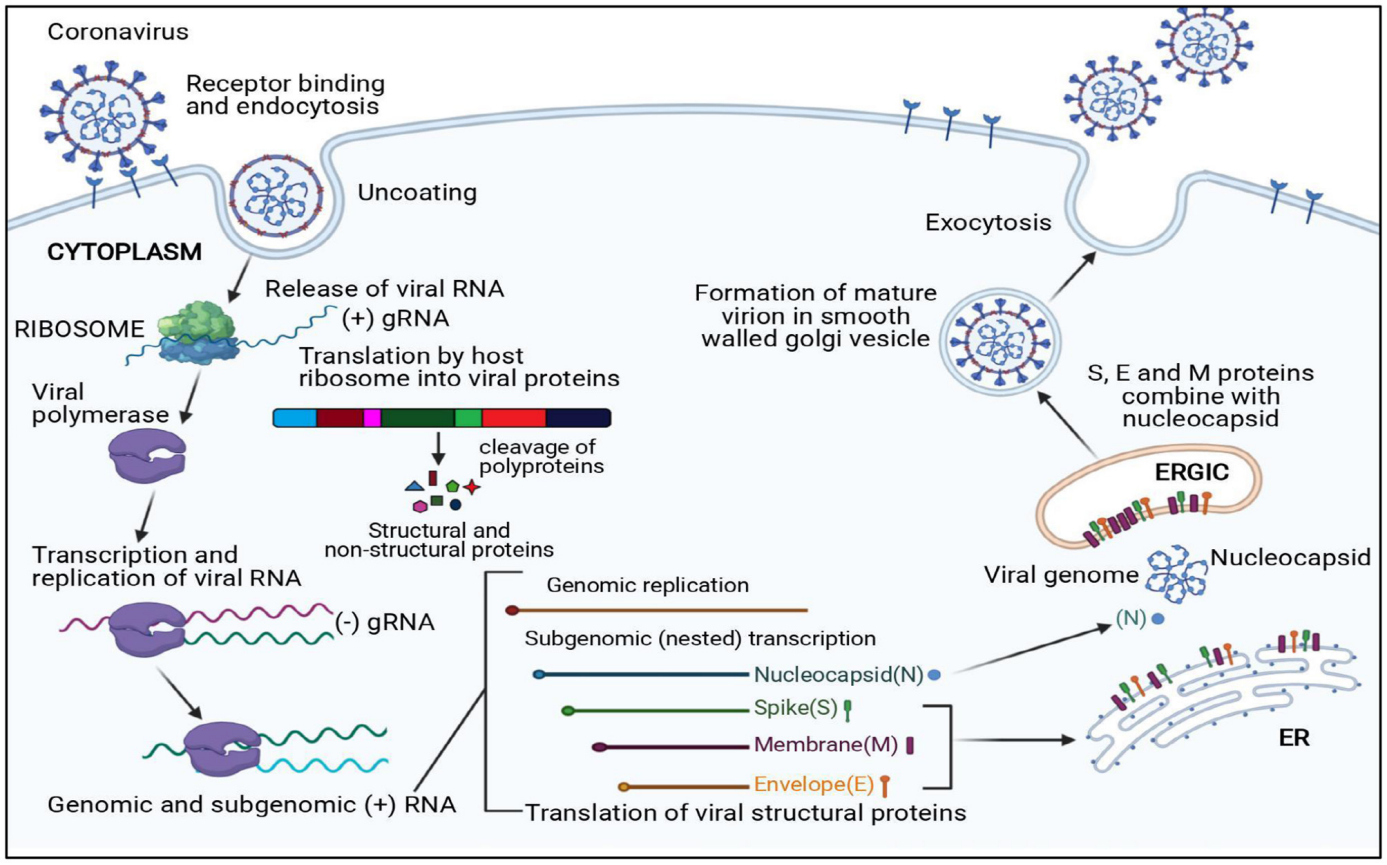
Replication cycle of coronavirus. N, Nucleocapsid; S, Spike protein; M, Membrane protein; E, Envelope protein; ER, Endoplasmic reticulum; ERGIC, Endoplasmic reticulum–Golgi intermediate compartment.

The viral replication cycle of all the CoVs is confined to the cytoplasm (Figure 2); additionally, murine CoVs can also replicate in enucleated cells (34–36).

The first essential step of the viral replication cycle is host cell receptor recognition by S protein and its attachment to the cell. The membrane fusion event results in penetration of virion aided by “fusion peptide,” which is exposed after variable rearrangement of S protein initiated by proteolytic cleavage of spike protein and acidic pH. This is followed by synthetic events such as translation of replicase gene from viral genome and formation of polyproteins, transcription, and RNA synthesis. After replication and RNA synthesis, the S, E, and M viral structural proteins are translated and inserted into the endoplasmic reticulum. Both M and E proteins function together to form envelope and virus-like proteins. The N protein binds to viral RNA and is later accompanied by M protein, which keeps the N protein and RNA complex stable. This interaction facilitates the assembly of virus particles on the membrane of the endoplasmic reticulum–Golgi intermediate compartment and initiates the budding process. Mature virions formed within the membrane-bound vesicles are released by exocytosis. The *viroporin* of E protein with ion channel activity promotes virus release by altering cell secretory pathways.

## Genome Organization and Role of Spike Protein in Evolution of Coronaviruses

The organization of large 20–32-kb size, capped and polyadenylated genome of CoV contains seven common genes in the following order, 5′-leader-untranslated region (UTR)-replicase-Spike (S)-Envelope (E)-Membrane (M)-Nucleocapsid (N)-3′ UTR-poly (A) tail.

**Figure 3:**
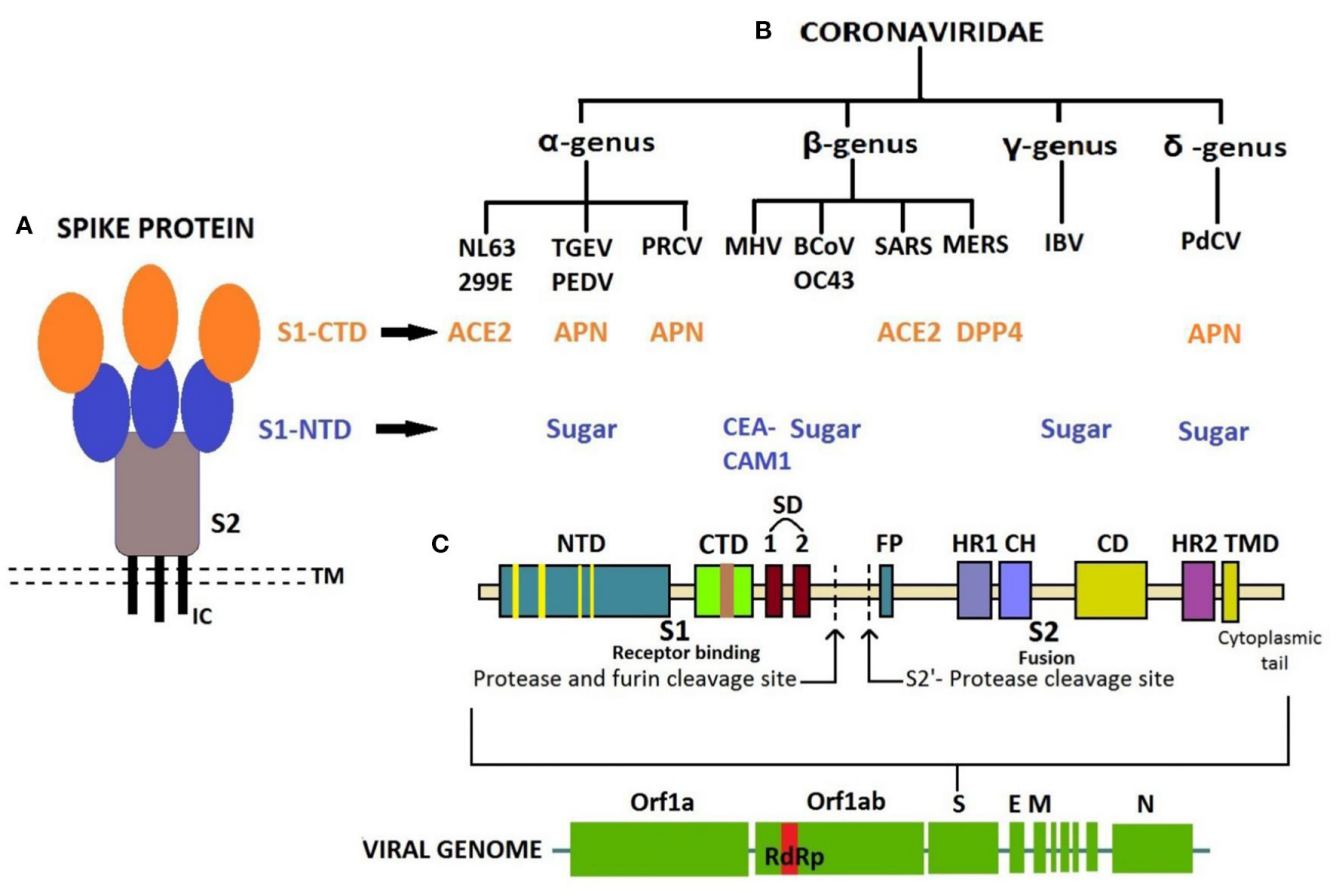
**(A)** Structure of Spike protein. **(B)** Classification of coronaviruses based on host cell recognition pattern by spike protein. **(C)** Genome organization of coronavirus and single S protein, from N- to C-terminus in left-to-right orientation. N-terminal domain in blue with receptor-binding motif (RBM) in yellow; C-terminal domain in green with RBM in brown. CTD, C-terminal domain; NTD, N-terminal domain; TMD, Transmembrane domain; IC, Intracellular tail; ACE2, Angiotensin-converting enzyme 2; APN, Aminopeptidase N; CEACAM1, Carcinoembryonic antigen-related cell adhesion molecule 1; DPP4, Dipeptidyl peptidase 4; SD, Subdomain; FP, Fusion peptide; HR1, Heptad repeat 1; HR2, Heptad repeat 2; CH, Central helix; CD, Connector domain.

The receptor-binding S1 subunit of spike proteins contains two distinct domains, the N-terminal domain (S1-NTD) and the C-terminal domain (S1-CTD). These domains recognize at least four protein receptors and three sugar receptors of the host cell (Figure 3) and, thus, can form the basis for classification according to the host cell recognition pattern (35, 37). The open reading frame (ORF) 1a/b encompasses a much larger section, i.e., the initial two-thirds of the genome encoding two viral replicase polyproteins—pp1a and pp1ab. These polyproteins are then further processed into 16 non-structural proteins (nsp1–16) by viral proteases and assemble to form a membrane-associated viral replicase–transcriptase complex (38–40). These are conserved among the subgroups of CoVs and thus share their relative position in the genome (41–44). Structural and some accessory proteins occupy only the last third of the coding capacity of the genome (45, 46) despite their range of complexity and function (40, 47).

Divergence in the sequence and motifs or residues of these proteins among CoVs may corroborate in classifying them in groups. Many researchers have demonstrated the phylogenetic relationships among their genomes based on the analysis of ORF1b replicase protein, 3C-like proteinase, polymerase, and structural proteins, which confirm the presence of different CoV group clusters (48–51). For instance, pairwise alignments of the corresponding ORFs and proteins of HCoV-OC43, bovine CoV (BCoV), PHEV, ECoV, and MHV suggest sequence similarity among them under the β-CoV group (50, 51). ORF8, a highly variable accessory gene and showing structural changes, plays a significant role in the evolution of SARS-related CoVs (52). The absence of a 29-nucleotide (nt) sequence in ORF8 and the presence of characteristic motif of single-nucleotide variations located in the S gene were observed in later phases of the SARS-CoV outbreak in 2002–2003 (53, 54).

The interaction between receptor-binding domain (RBD) and its host cell receptor helps in determining the CoV host range and cross-species infection (55, 56). This is dependent on the topology of RBD, its receptor-binding motif (RBM), and virus-binding motifs on specific proteins or sugars that complement each other in shape and chemical details. Both the distinctive domains, S1-NTD and S1-CTD of receptor-binding S1 subunit of CoV spike protein, can function as RBDs (57).

CoVs have been shown to mutate with high rate and recombination frequencies in their RNA genome (~10^−4^ nucleotide substitution/site/year). The mutations in the RBD of the spike gene are of significance, along with errors in the O-linked glycans and furin cleavage site, enzymes such as replicase and RNA-dependent RNA polymerase (58–60). The Indian SARS-CoV-2 Consortium on Genomics working on genome sequencing of new variants of SARS-CoV-2 has been reporting mutations and deletions in the amino acid sequence of spike protein since the SARS-CoV-2 pandemic. These mutational changes have led to the emergence of new double and triple mutants, alpha, beta, gamma, and delta variants with the capability of immune escape, increased virulence, transmissibility, and changes in clinical disease presentation (61).

The S1 subunits from the same genus share significant sequence similarity, whereas those from different genera have little sequence similarity (57). However, the speculation on members placed in different genera identifying the same receptor protein or those in the same genera identifying different receptor proteins still holds, despite evidence of a common evolutionary origin for the S1 subunit. The studies reveal that viral RBDs of CoVs of the same genus have a conserved CTD core structure but marked structural variations in their RBMs that enforce recognition of different receptors (62–65). Also, several other studies demonstrate that the viral RBDs of CoVs from different genus can bind to the same protein receptor due to the presence of a common virus-binding hot spot on the protein (62, 66). Thus, the data mentioned earlier provide an insight into an extensive divergent evolution of CoV S1-CTDs (67).

The crystal structure of β-genus MHV S1-NTD complexed with mouse CEACAM1 protein and BCoV S1-NTD with a sialic acid (SA) named Neu5, 9Ac2 (5-N-acetyl-9-O-acetylneuraminic acid) have the same structural fold in its core structure as for human galectins (galactose-binding lectins). Nevertheless, the BCoV S1-NTD is determined by its sugar-binding site instead of protein due to subtle changes in the conformations of their RBM loops and mutagenesis (68). This suggests that the ancestral CoVs inserted the host galectin gene into 5′ end of their spike gene, which resulted in CoV S1-NTD. After that, CoV S1-NTDs underwent divergent evolution in α, β, and γ genera, out of which S1-NTDs of β-genus BCoV, α-genus transmissible gastroenteritis virus (TGEV), and γ-genus infectious bronchitis virus (IBV) evolved their lectin activity and specificity for a different sugar receptor other than galactose. On the other hand, β-genus MHV S1-NTD subsequently lost its lectin activity and evolved specificity for a novel protein receptor, CEACAM1 (62). The S1-NTD of newly identified porcine delta coronavirus (PdCoV) (1, 69) shares a similar structure as α-CoV, β-CoV, and host galectins; thus, it recognizes sugar as its potential receptor and binds to sugar moiety of mucin to facilitate initial viral attachment, whereas the S1-CTD has the same structural fold as α-CoV S1-CTDs, but it differs from that of S1-CTDs of β-CoV (70). The PdCoV S1-CTD has a significant affinity for pig cells known to express aminopeptidase N (APN) as efficiently as TGEV-S1. Therefore, the porcine APN acts as a functional cross-genus receptor for both enteropathogenic PdCoV and TGEV for cellular entry (71). Such similarities suggest a close relationship between PdCoV, α-CoV, and β-CoV evolutionarily; however, PdCoV belongs to Deltacoronavirus owing to its genomic similarities with the avian species suggesting an ancestral avian origin (24).

The evolution of the spike protein of CoVs has also been proposed to help the virus in surviving against host immune response similar to the influenza virus (72, 73). The S1-NTDs of CoVs have also evolved the ceiling-like structure on top of its core to protect these sites and to evade the immune surveillance by the host immune system (74). As per the location of S1-NTDs and S1-CTDs on spike protein, the tips with S1-CTD are the protruding region and most exposed directly to the host immune system and therefore evolves at an increased pace to combat the host immune surveillance. Based on the immune pressure and different receptors (some of which are still unidentified) recognized by different animal CoV groups, it is clear that the S1-CTD exhibits a common evolutionary origin and has undergone divergent evolution. Moreover, the monoclonal antibodies directed against spike protein demonstrate common antigenic determinants for β-CoVs, especially the members of subgroup embecovirus, i.e., BCoV, PHEV, and HCoV-OC43, which corresponds to a close antigenic relationship (75, 76). This can put forward some hypotheses concerning the origin of β-CoV members, adaptation to a human host, and recombination events leading to novel CoVs with different species specificity responsible for emergence.

## Topology and Properties of Other Important Structural Proteins

**Figure 4:**
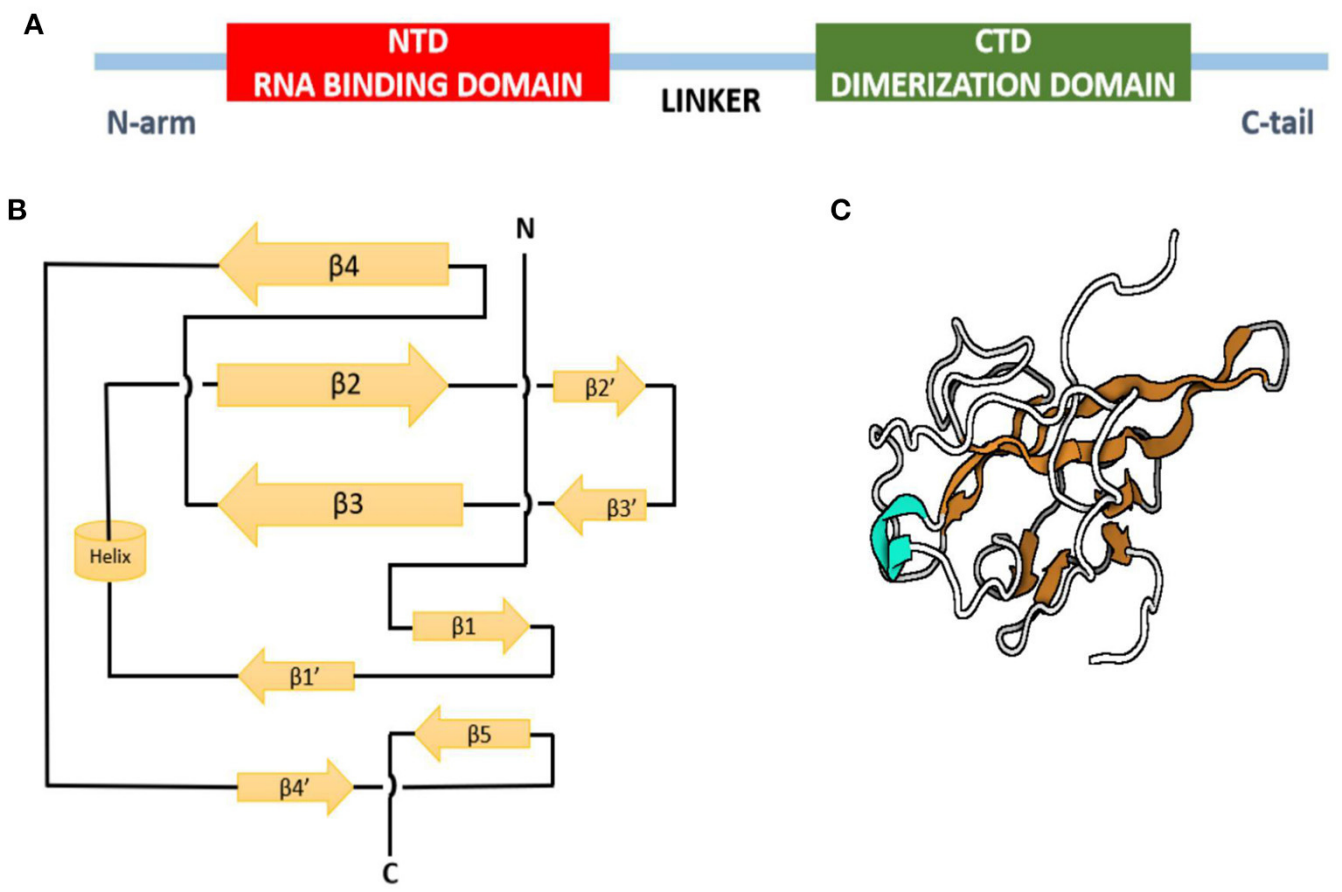
Nucleocapsid protein of infectious bronchitis virus. **(A)** Domain structure of N protein. **(B)** Topology diagram of N protein. **(C)** Three-dimensional structure of N protein. Beta strands in orange, and helix is shown in cyan.

The crystal structure of the N-terminal domain of nucleocapsid protein of MHV shares a similar topology structure with that of SARS-CoV and IBV containing five short β-strands (arranged as β4-β2-β3-β1-β5) across a U-shaped β-platform (Figure 4) but differs in its potential surface, indicating a possible varied RNA-binding module (77). The three residues, Arg-125 and Tyr-127 on the β3 strand and Tyr-190 on the β5 strand, provide a key role in transcriptional regulatory sequence RNA binding and helix destabilization essential for replication. These residues are totally invariant in betacoronavirus N proteins and incisively occupy analogous positions on the fold of each NTD, therefore likely to define similar RNA binding grooves between them (78). On the other hand, the sequence comparison of the C-terminal domain of N protein, also referred to as the dimerization domain as its residues form homodimers and homo-oligomers (oligomerization) (79, 80), shows that the domain is conserved at least among the alpha, beta, and gamma groups of CoVs, suggesting a common role for this domain.

The positively charged groove formed by the presence of the eight positively charged lysine and arginine residues of CTD is similar in SARS-related viruses and IBV-N CTD, except that the positively charged surface area in the SARS-CoV is larger than IBV (81) due to the absence of two lysine residues and the presence of additional negatively charged residues in the IBV N protein (82). Oligomerization and interaction of proteins with the viral genome is required for packaging of the genome by CoV N proteins to form ribonucleoprotein complexes for viral assembly (83). These functions of N proteins are performed similarly by SARS-CoV, IBV, and MHV. Thus, the overall similarity in the topology of the NTD and CTD domains of the N protein from SARS-CoV, IBV, and MHV fortifies a conserved mechanism of nucleocapsid formation for CoVs (74).

The primary sequence of the E proteins shows large variations in sequence and size among the groups with <30% identity and conserved membrane amino acid residues (84). Multiple membrane topologies of E proteins have been determined between different CoVs depending on the level of protein expression and oligomerization (84). The experimental studies have shown that the IBV E protein exhibits topology of cytoplasmic C-terminus while N-terminus in the lumen of the Golgi complex (85). Conversely, the TGEV E protein has a luminal C-terminus and N-terminus located cytoplasmically (86, 87). The CoV E proteins of only IBV, SARS-CoV, and MHV function for palmitoylation, i.e., modulation of protein–protein interactions, subcellular trafficking of proteins across the membrane, and membrane anchoring (88–91). A protein-binding motif located at the end of the C-terminus is highly conserved in α- and β-CoVs and is not found in the γ-CoVs (92).

The primary M protein sequence varies, although the secondary structures and an amphipathic region of the transmembrane domain are also conserved in almost all the members of the family (93). The type of glycosylation in the M protein of α- and δ-CoVs is N-linked, whereas O-linked glycosylation is found in the β-CoVs, but it is not critical for the viral assembly (19, 94–96).

## Hemagglutinin-Esterase Glycoprotein in β-Coronaviruses

**Figure 5:**
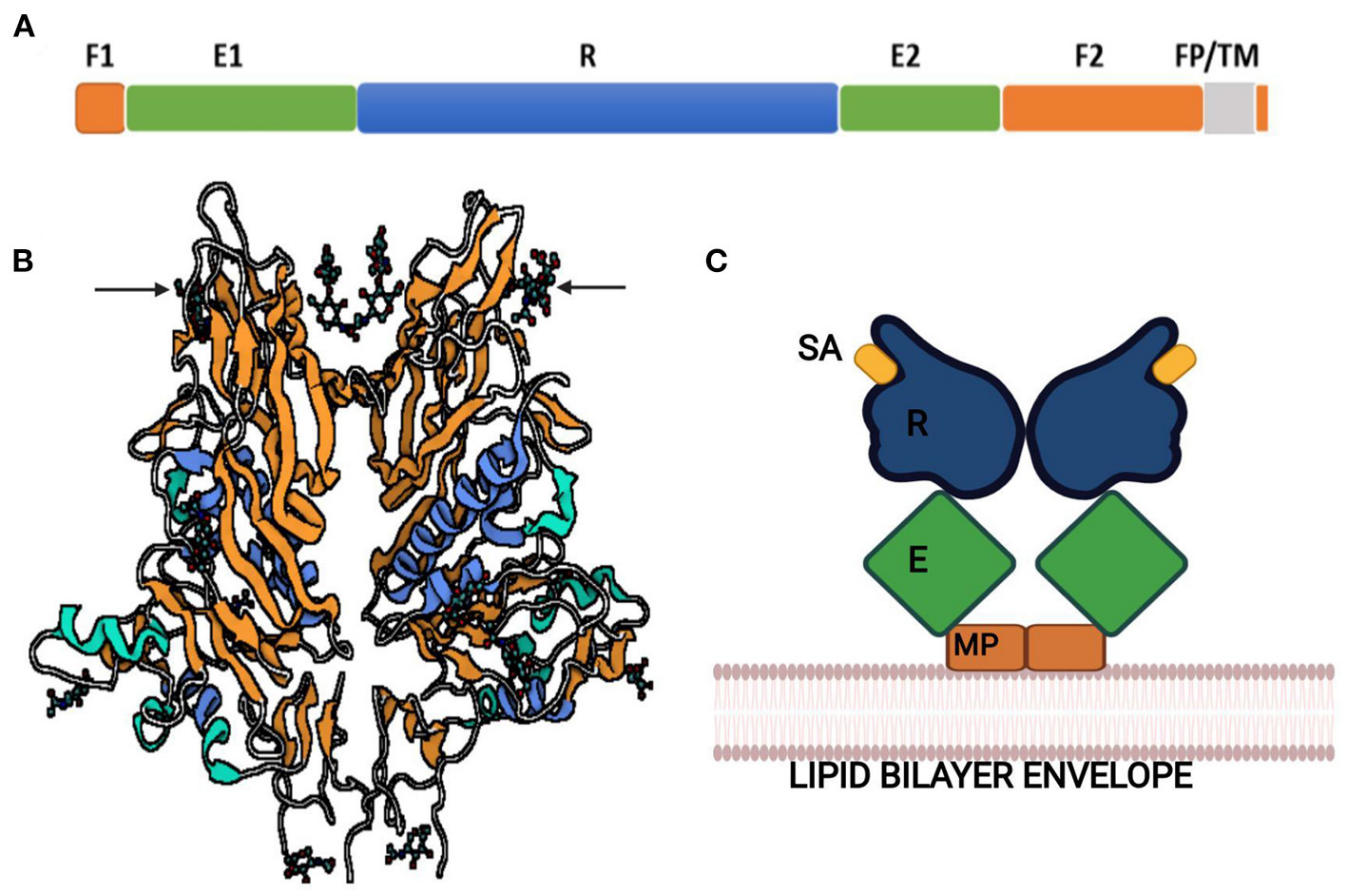
Hemagglutinin-esterase protein of bovine coronavirus. **(A)** Linear order of sequence segments HE protein. F1 and F2, Fusion domains; E1 and E2, Enzyme domains; R, Receptor domain; FP, Fusion peptide; TM, Transmembrane domain. **(B)** Ribbon representation of HE protein. Lectin domain in orange, esterase domain in blue, and structures shown by arrows are sialic acids (sialic acid-9-O-acetylesterase) bound to lectin domain. **(C)** Schematic illustration of HE dimer. SA, Sialic acid; R, Receptor domain; E, Enzyme domain; MP, Membrane-proximal domain.

The hemagglutinin-esterase (HE) gene is exclusively present in members of β-CoVs. CoV HE proteins were firstly identified from the PHEV, BCoV, and HCoV-OC43 bearing SA-9-O-acetylesterases similar to a hemagglutinin-esterase fusion protein of influenza C virus (97). The HE gene of CoV shares 30% sequence identity with the subunit of a HE fusion protein and has been found to be acquired by independent, non-homologous recombination events or evolutionary trajectories between influenza virus, torovirus, and CoV (98–100). All the CoV HEs are O-acetyesterases, whereas BCoV and HCoV-OC43 have dual activity of both hemagglutination and acetylesterase (101). Both these CoVs can agglutinate chicken erythrocytes, whereas purified HE protein of BCoV only agglutinates Neu5, 9Ac2-enriched erythrocytes of rodents. On the contrary, purified S glycoprotein can agglutinate chicken erythrocytes (102). This indicates that the major hemagglutinin is the S protein that also acts as the major SA-binding protein. The function of the hemagglutinin-esterase enzyme relies on the distinctive carbohydrate-binding domain as lectin and receptor-destroying enzyme domain (Figure 5).

HE protein with these domains and SA-O-acetylesterase activity mediates viral entry with S glycoprotein and attachment to the O-acetylated SA receptors on the host cell. The acetylesterase of murine CoVs prefers to esterize 4-O-acetyl-NeuAc and thus has different substrate-binding specificity than BCoV and HCoV-OC43, which targets 9-O-acetyl-SA (103). The combined activity of S glycoprotein and HE is specific for human CoV attachment to SA-associated receptors on the host cell (104), but the role of HE protein in HCoV other than HCoV-OC43 is not known much. However, the HE protein of SARS-CoV-2 also acts as the classical glycan-binding lectin and receptor-destroying enzyme and may show evolutionary adaption toward recognition of O-acetylated SA and virus entry for viral–host interaction (105).

## Bovine Coronavirus (BCoV)

BCoV belonging to genera Betacoronaviruses subgroup A along with swine HEV, canine respiratory CoV, feline enteric CoV, human CoV-OC43, and HKU1 is associated with three major clinical syndromes: neonatal calf diarrhea, hemorrhagic winter dysentery in adult cattle, and respiratory infections in cattle of different ages (106–118). It is an important livestock pathogen having an economic impact on the cattle industry worldwide (119). It is a leading cause of enteritis in combination with other enteric bacterial, viral, parasitic, and protozoal pathogens and is also found to be involved in the bovine respiratory disease complex in feedlot cattle since its discovery in 1993 (106). The host range includes all breeds of cattle and wildlife ruminants. The SA receptor for BCoV reflects wide tissue tropism due to the presence of sugars in abundance for interaction between viral spike glycoprotein and a specific carbohydrate receptor (102).

The BCoV variants, which are genetically and/or antigenically related, have also been isolated from other animal species along with humans representing a similar respiratory and enteric form of the disease (9, 120–123). Despite antigenic variations between different strains and interspecies transmission, only a single serotype is evident (124).

### Epidemiology

Infection is probably distributed worldwide—Africa, Asia, Europe, Oceania, and North and South America (125). The virus is shed in feces and nasal secretions predominantly. A study on naturally and experimentally infected animals revealed an excess of virus load isolated from nasal swabs and massive replication in airways, whereas the fecal shedding started later (126). It is readily transmitted by the feco-oral or respiratory route indirectly and directly by direct contact or aerosols on farms, maintained by a clinically normal cow and calves where adult animals can act as carriers. Calves of 1 week to 3 months of age are highly susceptible due to inadequate maternal antibodies. The adults are usually subclinically affected, and the virus may be excreted intermittently at low titer (127).

### Pathogenesis

The virus replicates in the epithelial cells of the upper (nasal turbinate, trachea) and lower (terminal bronchioles, lungs) respiratory tract: intestinal tract mainly along the lining of villi and crypts of epithelial cells. These cells are capable of resisting viruses and have the ability to replace the damaged cells (106), and thus, calves may recover from infection. The replication results in the destruction of mature absorptive cells lining the villi and mucosal surface in the large intestine, necrosis of cells in mesenteric lymph nodes and payer's patches, and subsequently viremia (125). This diminishes the absorption in the gut, failure to secrete digestive enzymes impairing the glucose and lactose metabolism and causing malabsorptive diarrhea. Pathological lesions such as marked intestinal hemorrhages and extensive cell necrosis within crypts are observed, whereas lesions of the respiratory system include hemorrhages, atelectasis, intestinal pneumonia, and emphysema (128).

### Clinical Features

Infection in calves depends on age and their immune status. Coinfection with *Campylobacter jejuni*, enterotoxigenic or enteropathogenic *Escherichia coli*, and Rotavirus may expedite the severity of the disease. The morbidity rate is 20–100% in affected animals, but mortality is 1–2% depending on the level of maternally or actively derived antibodies and severity of dehydration (119). The incubation period in calves is 24–48 h, and the clinical signs include profuse diarrhea, which subsequently results in dehydration, acidosis, and death in uncontrolled cases (123). In adult animals, the incubation period is 2–7 days and is the cause of acute sporadic enteritis prevalent during winter months, thus named winter dysentery. The disease is characterized by explosive, often hemorrhagic diarrhea, anorexia, emaciation, and unthriftiness along with decreased milk production along and frequent respiratory signs including fever, rhinitis, dyspnea, rales, pneumonia in 2–6 months old calves, and serious respiratory distress followed by death (125). The pneumotropic strains of the virus in adults are the precipitating cause of the bovine respiratory disease complex that exacerbate the fatality when manifested by superimposed environmental or managemental stress.

## Infectious Bronchitis Virus (IBV) in Poultry

Infectious bronchitis is an acute, highly contagious disease responsible for the economic impact on the poultry industry. The majority of CoV of avian species are classified into genera gamma- and delta-CoVs, within which IBV is of significance belonging to the gamma genus. Chickens and pheasants are the natural hosts but have also been detected in turkey, duck, guinea fowl, pigeon, peafowl, goose, teal, and partridge (129). IBV has a primary affinity for the respiratory system, accompanied by infection in the reproductive, renal, and alimentary systems. IBV occurs in various antigenic variants with a difference in virulence and tissue tropism as a result of mutations and recombination in its genome. The multiple serotypes of IBV based on S1 spike protein difference present a challenge in establishing an effective vaccination program, as cross-protection is found to be poor (130–132).

### Epidemiology

The distribution is worldwide, but some may have restricted geographical spread where different antigenic variants can co-circulate in a given region (133). It is of significant concern in poultry industries due to poor weight gains in broilers and suboptimal downgrading of egg production in layers (134). Birds of all ages are susceptible to the infection, but the severity and clinical signs may vary (135). In the acute phase of infection, IBV is copiously shed in respiratory secretions, tracheobronchial exudate, and feces and is spread by aerosols, ingestion of contaminated feed, drinking water with feces, and indirect transmission between birds at the farm over long distances through fomites (136). The vertical transmission is not clearly understood; however, the virus was isolated from day-old chicks and recovered from the semen of cockerels after inoculation (137, 138). The excretion and persistence of some strains of virus for a considerable time in target sites such as kidney or alimentary tract, particularly cecal tonsils followed by re-excretion, is suspected to be influenced by adverse environmental or changed physiological conditions, which suggests carrier state or latency (134). The strain of virus, route of exposure, age, diet, nutrition (level of calcium), and external factors such as cold stress in winter, poor ventilation, and coinfection with enteric bacteria provoke the disease; also, breed factor, such as some heavier birds become more susceptible, may be related to immune response (129). Although the morbidity rate is high as 100%, the mortality rate can vary from 20 to 30% and more depending on vast tissue tropism, secondary bacterial infection, and standards of management in an infected flock (139).

### Pathogenesis

The initial replication occurs in ciliated epithelial cells of the respiratory tract, which cause histopathological lesions mainly in the trachea, such as ciliary loss, desquamation, epithelial hyperplasia, edema, marked lymphoplasmocytic inflammation, and mononuclear and heterophilic cell infiltration of the submucosa, which resumes in 14–21 days after infection (140, 141). A succinct viremia within 1–2 days eventually leads to extensive spread to the reproductive system, kidneys, and intestinal tract, but the damage is minimal, and bursa of fabricius may be the cause of immunosuppression. The main attachment factor for IBV is the receptor-binding domain in S1 spike glycoprotein and SA glycans (142, 143) widely distributed in host tissues; thus, variation in the glycoprotein and glycans partly determine the virulence, tissue binding, and tropism. The gross pathological findings include congested respiratory tract with serous or catarrhal exudate in nasal passages, trachea, extrapulmonary bronchi, and air sacs. The main bronchi get blocked with caseous casts in young chicks, the probable cause of death. The epithelial cells of the oviduct, mainly the goblet cells, become cuboidal, hypoglandular oviduct, ovarian regression, and congestion; sometimes, the ova may rupture, resulting in free yolk in the abdominal cavity (144, 145). Extensive tubular degeneration, interstitial inflammatory response characterized by the pale, enlarged, or marbled kidney, ureters distended with deposits of urates, and large uroliths are seen in the chronic stage of nephritis (141, 146, 147). Certain IBV strains also induce pathological lesions in deep and superficial pectoral muscles, i.e., bilateral myopathy in broilers and breeders (148).

### Clinical Features

The incubation period is 18–48 h; the course of the disease lasts for 5–7 days and, in outbreaks, up to 14 days (146, 149). Chicks with an age of 2–6 weeks are severely affected, although birds of all age groups are susceptible. The main three clinical manifestations are respiratory, reproductive disorder, and nephritis (134). The most conspicuous clinical findings are the initial respiratory signs—gasping, tracheal rales, dyspnea, swollen sinus, conjunctivitis, profuse lacrimation, cellulitis of periorbital tissues, and coughing with or without nasal discharge. This is followed by lethargy, ruffled feathers, anorexia, rapid weight loss, stooped stance, scouring, excessive water intake, and characteristic wet litter implying nephritis. The reproductive disorder shows signs of rales followed by a marked decline in egg production up to 50–70%, usually within 8–12 days, which differs depending on the stage of lay at infection, hampering the hatchability rate (150). The external and internal quality of the egg is highly affected, exhibiting misshapen eggs, thin, soft, or no shell, ridging and distortions, watery albumen, which may resume within 8 weeks or more. It may also lead to permanent damage to immature oviduct resulting in so-called false layer syndrome, as the layers or breeders never resume the loss of egg production. The concurrent secondary infection with *E. coli*, avian mycoplasma species, etc., or nephropathogenic strain of IBV may expedite the infection causing air-sacculitis and interstitial nephritis (151). Chicks may die suddenly by occlusion in bronchi as a probable cause of death.

## Porcine Coronaviruses

### Transmissible Gastroenteritis Virus (TGEV)

Among few porcine CoVs known, the clinical disease is mainly associated with the TGEV. It is highly contagious among young pigs and is found to be a significant cause of economic loss more in breeding herds than the rearing and finishing herds, primarily due to piglet mortality (152). The porcine epidemic diarrhea virus (PEDV) is clinically similar but serologically unrelated to TGEV and comparatively spreads slowly in the herd (11). The porcine respiratory coronavirus (PRCV) is a non-pathogenic respiratory variant of TGEV (153, 154). It can cause subclinical mild respiratory disease and serologically cross-reacts with TGEV, but tests are available to distinguish them. These porcine CoVs are grouped in genus Alphacoronavirus; however, a new porcine delta-CoV genetically distinct from TGEV and PEDV associated with enteric disease in pigs has recently been found (155).

### Epidemiology

TGEV is reported all over the world, affecting the global pork industry (156). It spreads between and within farms by shedding infected feces for up to 2–3 weeks. The virus may also spread through fomites, aerosols at least for short distances, or mechanical spread by animals, insects, or birds, particularly starlings and in milk or feces to the piglets (157). The infection occurs throughout the year but mainly follows a seasonal pattern with a higher incidence in colder months (158). Infection results in two different clinical presentations: epidemic and endemic (159). In epidemics, when a virus enters a naive herd, pigs of all ages are affected, particularly the newborn piglets, whereas the infection is self-limiting in farrowing and finishing herds. The endemic disease is observed in farms after the epidemic phase due to incomplete all-in-all-out management or continuous movement of naive gilts in breeding farms. TGEV can end up showing mild disease, thus presenting high morbidity up to 100% in neonatal piglets but low mortality (11).

### Pathogenesis

The virus enters through the oro-nasal route and replicates in enterocytes of the small and large intestines. The replication causes shortening and blunting of villi, mainly in jejunum and ileum, due to the segmental nature of lesions followed by malabsorption, disruption of cellular transport of nutrients and electrolytes, and increased osmolarity, thinning of the gut wall, and diarrhea (160, 161). The crypts of epithelial cells usually remain uninfected; thus, recovery of function of villi is rather rapid. In neonates and piglets, a combination of these factors coupled with the slow regeneration time of epithelial cells results in death. TGEV has also been found to replicate in extra-intestinal tissues, including lungs and mammary gland, causing imprecise pneumonia and agalactia, respectively (162, 163).

### Clinical Features

The incubation period is short 12 to 72 h, i.e., up to 3 days (164). TGEV presents a mild disease except in piglets ≤3 weeks of age that may succumb to death and in sows infected at or near farrowing. Vomition is the initial sign in a non-immune herd followed by profuse watery diarrhea, rapid dehydration, weight loss, marked thirst, and agalactia with recovery within 5–10 days (11, 165). As the disease progress in unweaned piglets, feces often contain curds of undigested milk and may approach 100% mortality due to the slow replacement rate of villous cells. The course of disease in porcine CoV infections does not exceed 3–4 weeks normally due to rapid herd immunity; thus, the mortality is low, but morbidity is high (159). In some herds, the TGEV remains subclinical, although there may be short episodes of clinical reemerging infection particularly due to the purchase and replacement of breeding pigs and their litters.

## Feline Coronaviruses

Feline CoVs are classified into two biotypes based on the pathogenicity referred to as feline enteric coronavirus (FECV) and feline infectious peritonitis virus (FIPV) belonging to genus betacoronavirus and alphacoronavirus, respectively. Higher sequence similarity in both the biotypes indicates a close relationship but with distinct virulence properties (166–169). The FIPV is primarily observed in a cat population that is tenaciously infected with FECV (170, 171). These recurrent observations and animal experiments have led to the widely accepted theory of “internal mutation” that suggests an evolution of FIPV from non-pathogenic FECV by specific mutation(s) occurring in the viral genomes (172–179). The sequence differences in spike and membrane protein, mutations in theS1/S2 locus, furin recognition site, and disrupted NSP3c genes may further contribute to the risk of FIP in the individual (180). However, very less is known about the stage at which mutation(s) occur during the development of FIP. The FCoVs are further separated into two serotypes based on the serological properties of the virus (181). Some independent studies provide constant evidence that the emergence of serotype II viruses is *via* double homologous recombination between serotype I FCoV and canine enteric coronavirus (CCoV) (170, 182–186). Both serotypes I and II can cause FIP and clinically inapparent FECV infections.

### Epidemiology

FCoV infection is widely disseminated in the domestic and wild feline population. The seropositivity varies from 20 to 60% and approaching up to 90% in domiciled cats, multi-cat households, catteries, and animal shelters, according to the global data (171, 187–189). The serotype I FCoVs are mostly responsible for natural infections (187, 190–192). Serotype I FCoV strains are vastly isolated from the United States and Europe (80–95%), whereas serotype II predominates in Asia in up to 25% (192–196) analysis. The seroprevalence studies had demonstrated high incidences and seropositivity in cats from 3 months to 3 years old and in adult individuals (190, 197, 198). There is no significant difference in seropositivity related to sex and breed of cats; however, genetic predisposition can affect the reproductive condition, hereditary factors and systemically manifest the disease (171, 199). FIPVs in animals are less likely to be transmitted horizontally, and infection due to contact with feces from diseased cats (172, 178, 200–202) is thought to be limited. However, immunosuppression favored by stress or coinfections with feline immunodeficiency virus and feline leukemia virus may trigger the progression of FIP in some cases (171). In contrast, FECV is highly contagious and transmitted horizontally through the fecal–oral route (167, 171, 188). The infected cats can continually shed FECVs in their feces for a longer period and even in postinfection, which may last for several months but with low virus load (167, 203).

### Pathogenesis

The main site of FECV replication is the apical epithelium of the villi from the lower portion of the small intestines extending to the cecum (167, 203). In addition, the viral RNA can be recovered from blood and different tissues as well, suggesting the capability of FECV to infect peripheral monocytes, albeit less efficiently (179, 203–207). FIPV presents an altered cell tropism and infects both monocytes and macrophages (170, 206, 208, 209). The distribution of macrophages in the body results in viral dissemination from the intestine to the spleen, liver, and central nervous system. Thus, it is considered as an immune complex disease involving activation of these cells (210) and expression of tumor necrosis factor-α, interleukin-1β, adhesion molecules, matrix metalloproteinase-9, vascular endothelial growth factor, vasoactive amines, and inflammatory mediators (210–217). These factors, along with less susceptible leukocytes activated by an unknown mechanism during FIPV infection, induce capillary endothelial cell retraction, increased vascular permeability, and hence protein-rich effusion in body cavities (218, 219). Therefore, the FIPV infection is characterized by fibrinous and granulomatous serositis, protein-rich serous exudates in body cavities, and/or pyogranulomas (213, 220–223).

### Clinical Signs

The infection caused by FECV remains persistent and asymptomatic and/or induces mild and transient diarrhea and occasionally causes severe enteritis (224). Feline infectious peritonitis is an immune-mediated, systemic, and fatal disease (190). The infection can be clinically distinguished into three forms based on the presence or absence of protein-rich effusions in the pleural and abdominal cavities—wet (effusive), dry (non-effusive), and a combination referred to as mixed form (171, 188, 213, 225, 226). The clinical progression of the disease is believed to be dependent on the host cellular and humoral immune responses. The wet form is associated with weak cellular but robust B cell responses, whereas the dry form is caused by strong T cell immune responses (171, 188). It has been observed that wet form is more prevalent in natural infections than other forms and frequently develops in the terminal stage of dry form resulting in subsidence of the immune system (171, 188). The wet form is characterized by abdominal, thoracic, or pericardial effusions leading to fluidic waves in the abdomen, visceral and omental adhesions or enlarged mesenteric lymph nodes, dyspnea, or tachypnea, cyanotic mucous membranes, and muffled sounds in the lungs and heart. The dry form is characterized by granulomatous changes in several organs, including the central nervous system and the eye. The ocular lesions include white sheathing of retinal vasculature, mild uveitis, keratin deposition in the cornea, and hemorrhages in the anterior chamber and retina. In cats with FIP, the neurological signs are variable and can induce multifocal lesions. Ataxia with subsequent seizures, tremors, nystagmus, incoordination, and hyperesthesia is the most common clinical sign. When FIP lesions are associated with cranial nerve, visual impairment and loss of menace response are observed, whereas lameness or paresis can be seen in peripheral nerve involvement.

## Canine Coronaviruses

CoVs of Canidae family fall in two groups— CCoV in group 1 alphacoronavirus and canine respiratory coronavirus (CRCoV) in group 2 betacoronavirus. CCoV has been described since 1971 (227) and exists in two closely related serotypes—CCoV-I and CCoV-II based on random point mutations and recombination associated with the spike protein and distinct serological properties (182). Type I and II CCoVs and FCoVs have been proposed to be closely related based on their evolution through recombination events from a common but unknown genetic source (186, 228). CCoV-II can be further classified into CCoV-IIa and CCoV-IIb as a result of the recombinant origin of CCoV with NTD of spike protein homologous to TGEV (229, 230). A novel CCoV with CCoV-I- or FCoV-I-like NTD was discovered in 2014 and was referred to as CCoV-IIc but has not been classified into any clade yet (231–233). CCoV generally causes mild and self-limiting diarrhea with low mortality and high morbidity (234, 235). Virulent and pantropic strains of CCoV causing severe enteric and fatal systemic diseases in the absence of coinfection with canine adenovirus type I and canine parvovirus type 2 have also been reported (236–244). Therefore, CCoV is now considered a significant pathogen in the dog population due to its ability to evolve into variants with altered tissue tropism and pathogenicity.

CRCoV was newly recognized in 2003 from the tracheal and lung samples of dogs facing enzootic respiratory disease (9). It is one of the members of multiple etiologies causing canine infectious respiratory disease along with canine parainfluenza virus, canine *adenovirus (CAV) type 2, canine herpesvirus and canine influenza virus, Bordetella bronchiseptica, Streptococcus equi* subsp. *zooepidemicus*, and *Mycoplasma* spp (245, 246). CRCoV was found to carry an additional gene encoding for HE protein; thus, it shows high sequence identity up to 98% with BCoV and human CoV OC43 along with similarities in spike protein and polymerase gene sequences (9, 247).

### Epidemiology

Both CCoV and CRCoV are common infections of the canine population with worldwide distribution (121, 248–258). Single, as well as multiple infections, have been reported with more than one genotype of CCoV that indicates co-circulating of CCoV–I, CCoV-IIa, and CCoV-IIb strains in prevalent regions (259). Canine CoVs are highly prevalent in dogs living in dense populations such as shelters, kennels, or grouped environments and thus exhibit rapid transmission through feco-oral and naso-oral routes (260). Dogs are likely to act as clinically normal carriers maintaining the infection in the canine population due to long time shedding of CCoV after postinfection and clinical resolution (261, 262). Apart from the domestic dogs, canine CoV infection has also been reported in foxes, wolves, and raccoon dogs (263, 264). The infection occurs throughout the year in dogs, whereas CRCoV is frequently detected during the fall to winter months (265). Canine CoV infections can occur in all age groups, significantly more in young puppies for CCoV in contrast to CRCoV, which is most prevalent in dogs more than 1 year age (254, 255).

### Pathogenesis

The pathogenesis of CCoV is similar to that of other enteric pathogens. It replicates in the apical and lateral mature epithelial cells of intestinal villi resulting in villous atrophy and consequently malabsorption and diarrhea (233). The severe form of enteritis represents gross pathology as moderate, diffuse, segmental hemorrhagic and necrotic enteritis, ileo-cecal intussusception, along with infiltration of lymphocytes and plasmacytes (244). Systemic infection caused by pantropic CCoV produces lesions in several organs, including infarction in the renal cortex, fibrinopurulent bronchopneumonia, fatty change in the centrilobular zone of the liver, multifocal hemorrhages in the spleen, and depletion of gut-associated lymphoid tissue (266).

The pathogenesis of CRCoV is mainly associated with the trachea, nasal cavity, and nasal tonsil with less severity in the lower respiratory tract. It causes distortion of the ciliated respiratory epithelium and infiltration of inflammatory cells resulting in failure to clear the particulate matter in the lungs, bronchi, etc., (267). The virus has also been isolated from the colon, mesenteric lymph nodes, and spleen (121, 257). This suggests dual tissue tropism similar to BCoV, although the ability to replicate in tissues other than the respiratory tract needs further investigation. Furthermore, the possible interaction of multiple pathogens during the canine infectious respiratory disease complex needs to be considered as contributing factor in the pathogenesis of CRCoV.

### Clinical Signs

CCoV generally causes mild and self-limiting diarrhea in dogs. The severity of enteric disease increases when infected with multiple pathogens or pantropic strains (CCoV-IIa biotype) of CCoV. The clinical signs thus include gastrointestinal distress, hemorrhagic diarrhea, along with neurological signs (268, 269). The infection with CRCoV exhibits common and mild clinical signs associated with the upper respiratory tract, including sneezing, coughing, and nasal and ocular discharge (267), which may progress to bronchopneumonia and multisystemic illness depending on the involvement of other organs.

## Diagnosis of Animal Coronaviruses

Enteric and respiratory infections of CoVs are mainly associated with the shedding of the virus through feces and nasal secretions, respectively. The clinical samples for diagnosis include feces, intestinal contents, nasal secretions, tracheobronchial lavage fluids, and postmortem specimens comprising of nasal, pharyngeal, tracheal, lungs, and tissues from different regions of the gut focusing primarily on the distal small intestine. In addition, trachea, kidney, proventriculus, tonsil, and oviduct specimens for IBV and aqueous humor, whole blood and fine-needle aspiration, a biopsy of the liver, spleen, and mesenteric lymph nodes for FIP are obtained for diagnosis depending on the clinical presentation of the animal. Direct detection of virus in clinical samples by transmission electron microscopy, immunofluorescence, immunoperoxidase, or immunohistochemical staining of tissues using hyperimmune antiserum or monoclonal antibodies provides definitive diagnosis (171, 213, 221, 270–284). Immunohistochemistry using an antibody directed against BCoV can also help in detecting CRCoV (285). The laboratory tests using effusions have more diagnostic value than blood tests for FIP (286, 287). Cytological and macroscopic examination, along with cell count and biochemical properties of effusions, can be carried out for differential diagnosis (283, 286, 288–290). A simple, quick, and inexpensive “Rivalta's test” with more than 90% sensitivity and 66–81% specificity for differentiating transudate from an exudate can be useful to exclude FIP and rule out other causes for the effusions (287, 291).

Tracheal organ culture, McClurkin swine testicle (ST) cell line, human rectal tumor HRT-18 cells, Vero, and other cell lines derived from specific host species can be used for primary and secondary virus isolation and propagation favoring syncytia, plaque, or cytolysis induction (111, 112, 116, 118, 231, 291–297). To improve the detection of the typical cytopathic effect, it may require the addition of pancreatin or trypsin to the cell culture along with additional blind passages. The allantoic cavity of 9–10-day-old chicken embryo inoculated with IBV-infected material exhibits curling and dwarfism as characteristic IBV lesions observed in the embryo (298, 299). On the other hand, virus isolation of CCoV-I and CRCoV is often unsuccessful and, even if achieved, does not produce cytopathic effects (121, 183, 184).

Serological assays such as enzyme-linked immunosorbent assay (ELISA), virus neutralization test, immunofluorescence antibody test, rapid immunochromatographic tests, and blocking ELISA using monoclonal antibodies to differentiate between strains and serotypes of particular CoV are used (300–309). Serological tests are of limited value in IBV, CCoV, and FCoV, as they fail to discriminate between several serotypes. The BCoV antigens can also be used against canine sera instead of CRCoV in ELISA, serum neutralization, or hemagglutination inhibition test (9, 238, 256). The supernatant from CRCoV-infected cell culture was able to agglutinate chicken erythrocytes at 4°C, which means that hemagglutination assays can be optimized to detect CRCoV (310).

The highly sensitive molecular assays including serotype-specific reverse transcription-polymerase chain reaction (RT-PCR), nested PCR, real-time quantitative RT-PCR using conserved gene regions—UTR, N-gene, S1 gene, or HE gene (257), reverse transcription loop-mediated isothermal amplification assay, reverse transcription recombinase polymerase amplification assay, pan CoV RT-PCR are used providing high detection rates than other assays (176, 261, 286, 302, 311–339). The next-generation sequencing to decipher the whole genome within a short period is currently being used in advanced laboratories (340, 341).

## Novel Diagnostic Tools

With an increasing rate at which serious infections with new variant strains of human CoVs, especially SARS-CoV-2 spread, the need was felt to deploy rapid, accurate, and precise diagnostic tools for laboratory and point-of-care (POC)-based settings. This includes nucleic acid amplification testing such as the centralized laboratory-based real-time RT-PCR (the current gold standard for etiological diagnosis), rapid POC based tests such as lateral flow assays, rapid serological (antibody or antigen) tests, LAMP test, and serological assays such as ELISA and automated EIA (342–345). Some novel strategies for SARS-CoV-2 detection include CRISPR/Cas (reliable on-site diagnostic method)-based paper strip test, matrix-assisted laser desorption/ionization time-of-flight mass spectrometry combined with artificial intelligence, surface-enhanced Raman scattering spectroscopy, metabolomic approaches, aptamer (third-generation molecular probe)-based diagnostic tests, proteome microarray, optical biosensor, antigen-Au/Ag nanoparticle-based electrochemical biosensor, and surface plasmon resonance (346–350). Despite rapid refinement in existing tools and deployment of novel strategies, the advancement in diagnostics of animal CoVs comparatively fall behind and mostly rely on clinical diagnosis, detection, and titration of CoV particles (plaque assay, electron microscopy, and transmission electron microscopy), detection of CoV antibodies (ELISA and EIA), detection of CoV antigen (monoclonal- and polyclonal-based ELISA, rapid Ag detection tests, immunofluorescence, and immunochromatographic assays), and nucleic acid-based assays (RT-PCR, real-time PCR, and loop-mediated isothermal amplification-PCR). Although these molecular and serology-based methods provide accurate results, they require well-trained technicians, specific types of equipment, and ample time and effort and are not convenient for use on farms or by breeders. For this purpose, various studies have been undertaken to develop efficient and appropriate diagnostic tools for animal CoVs as has been seen with SARS-CoV-2. The diagnosis for a newly identified pathogen, porcine delta CoV, was made possible by developing a cost-effective fluorescent microsphere immunoassay as used for PEDV (351) that could detect antibodies against multiple target antigens of porcine delta CoV for better efficiency and sero-surveillance on a herd level (352). Recently, a europium (III) chelate microparticle-based lateral flow test strip was developed for identification and epidemiological surveillance of PEDV with high reliability and sensitivity (353). A novel multiplex PCR-electronic microarray assay for rapid and comprehensive detection of bovine respiratory and enteric pathogens, including BCoV, was also developed (354). A highly specific plaque reduction neutralization test was combined with two sensitive molecular methods: real-time RT-PCR and Sanger sequencing, to investigate SARS-CoV-2 infection in cats and dogs in Brazil (355). Similarly, a Luciferase Immunoprecipitation System assay using the fragments of spike protein and nucleoprotein as antigens was used to detect antibodies against SARS-CoV-2 in dogs and cats and MERS-CoV in dromedary camels and monkeys (356–358). For Luciferase Immunoprecipitation System assay, there is no need for a BSL-3 laboratory set up and species-specific labeled secondary antibodies for detection as required for other tests such as microneutralization, immunofluorescence assay, and the plaque reduction neutralization test. Moreover, many researchers have envisaged the use of lateral flow assays for diagnosis and pseudovirus-based neutralization assay for evaluation of antiviral mechanism for SARS-CoV-2 in animals as well (359, 360). As far as the specific and sensitive diagnosis of animal CoVs is concerned, currently available methods might not resolve the unprecedented challenges associated with CoVs due to rapid mutations, interspecies jumps, and the emergence of new variants. This warrants continual efforts to develop robust, sensitive, and rapid tests, POC devices, and multiple diagnostic techniques to achieve a cost-effective and multidimensional diagnostic efficiency for clinical and epidemiological investigations.

## Novel Vaccination Strategies

The CoV infection in animals is routinely managed by the whole cell-based inactivated or modified live vaccines (361–370). The inactivated vaccines give rise to a high titer of circulating antibodies, whereas modified or attenuated live vaccines provide stimulation of cell-mediated immunity and accelerate IgA response against local mucosal infection and thus are of more value to commercial farms. The protection against animal CoVs is mainly dependent on IgA production; thus, parental vaccination is not favored generally in many of these infections (362, 371–373). The intranasal vaccination in adult cattle entering the feedlots using live attenuated enteric CoV against diverse field strains causing BCoV infections has been suggested as an ideal strategy to develop a protective immune response at the site of entry (oro-nasal) of the virus (123). Currently, the attenuated live vaccines used in broilers, layers, and brooders provide relatively inferior protection due to the presence of several serotypes of IBV and poor cross-protection. Therefore, a protectotype vaccine strategy based on shared antigens among variants has been proposed based on knowledge about serotypes, immunity, and the prevalence of variant strains of IBV (374). These protectotypes are quite effective in inducing cross-protection against heterologous serotypes (375). The feline CoV vaccination may enhance the risk of immune-mediated FIP; therefore, activation of IgA response is more relevant than IgG production. The administration of a modified live intranasal vaccine (temperature-sensitive), which activates IgA in the oropharynx as a consequence of replication of a temperature-sensitive mutant of the FCoV strain, is found to be more effective in FCoV (362). Canine CoV infection is mild and self-limiting and thus discourages the wide application of vaccines even if inactivated and live attenuated vaccines have been successfully developed for CCoV (369). In an experimental trial, a beta-propiolactone-inactivated MF59-adjuvanted vaccine was developed against the CCoV/TGEV recombinant, which failed to prevent the shedding of the virus totally but was considered to be safe (376). Novel experimental vaccines for TGEV and PEDV such as plasmid-vectored DNA vaccines encoding S, N, or M (377, 378) and recombinant vaccines or vectored vaccines using engineered swinepox virus or porcine adenoviruses to deliver the TGEV spike protein (379) were also developed. These vaccines were efficient in inducing both a systemic and a local humoral immune response with sufficient neutralizing antibodies. An RNA vaccine derived from Venezuelan equine encephalitis replicon expressing the PEDV spike gene was also developed against PEDV infections (379). Subunit vaccines have also been developed in an effort to express the S1 domain of spike protein in Baculovirus, yeast, or plant-based delivery system and have been found to work well in pigs (380–382). The oil adjuvanted vaccine comprising of a solubilized cell extract of BCoV-infected cells overexpressing viral hemagglutinin of BCoV was developed by Takamura et al. (383). This induced high hemagglutinating antibody titers without any adverse effects and was suggested to prevent winter dysentery in dairy cows (383). It is possible to combine two or more immunogenic strains of protectotype candidate against animal CoVs in a single vaccine with combined benefits such as the stimulation of cell-mediated immunity and higher IgA production for local mucosal immunity. Animal CoVs such as PEDV and IBV undergo frequent genetic shifts, and as a result, the protection level remains low despite using innovative vaccine strategies. Also, the vaccination in animals provides relatively a short duration of protective immunity with low efficacy due to focus mainly on whole-cell preparations rather than specific proteins or antigens for developing vaccines. Hence, effective and safe vaccines providing long immunity in animals still remain elusive. The scientists working on developing veterinary vaccines must take a cue from innovative and successful emerging technologies being used in developing SARS-CoV-2 vaccines so that suitable prophylaxis also becomes available for animals.

## Interspecies Transmission and Zoonotic Potential of Animal Coronaviruses

### Interspecies Transmission

CoVs are capable of producing a broad spectrum of disease outcomes in mammals and avian species. These viruses are well-recognized to alter their tissue tropism and cross interspecies barriers to adapt to various ecological niches for abundance and persistence. The RBD of CoVs can recognize the same receptor present in multiple animal species. These virus–receptor interactions, evolutionary selection, and viral genetic diversity by frequent homologous recombination, inherent point mutations enable the virus to jump the species barrier and rapidly adapt to a new host species. It has been observed that CoVs, apart from infecting their primary host, do cause occasional infections in other animal species, *per se* dog and cat CoV, pig CoV, and TGEV can cross-infect, resulting in different disease outcomes suggesting host range mutants of an ancestral CoV. The BCoV is an excellent example that crosses interspecies barriers. Variants having genetic and antigenic relatedness with BCoV have been identified in the respiratory secretions of dogs infected subclinically (121–123), humans (120), and diverse groups of other domestic (124) and wild ruminant species such as camelids, waterbuck, sambar deer, white-tailed deer, and giraffe. Moreover, it can experimentally infect and cause enteric diseases in avian hosts, including turkey poults (384) that were found to transmit viruses to the control birds. These variant strains with established cross-species transmission are termed as Bovine-like CoVs (385) and classified as host range variants rather than a distinct virus. Furthermore, the BCoV has also been shown to procure new genes *via* recombination, i.e., acquisition of an influenza C-like hemagglutinin that may have a possible role in binding to different cell types (386).

An amino acid composition of APN receptor of human, feline, and porcine CoVs shows strong 78% identity. However, the APN receptor used by α-CoVs is species-specific, and the feline APN is a functional receptor for other members of α-CoVs, including FIPV and FECV, TGEV, canine CoV, and HCoV-229E. Due to this property of feline APN, cats can get infected by TGEV, CCoV, or HCoV-229E with or without developing symptoms. Similarly, the sequence comparisons reveal that TGEV has resulted from the host species jump of CCoV-II from dogs to pigs (40). The generation of less virulent porcine respiratory CoV from TGEV by spike gene deletion (153, 154) accounted for altered tissue tropism from the enteric to the respiratory system (387). The FIPV type II and CCoV-IIb strains from double recombination events between FIPV type I-CCoV and CCoV-II-TGEV, respectively, are also the results of genomic characteristics within CoVs for rapid adaption to novel CoV species suggesting coinfection in at least one host species.

A chimeric virus with spike protein of PEDV and backbone of TGEV was identified as a variant genotype of TGEV strain with unique deletions and distinct amino acid changes similar to PRCV, suggesting a recombination event between the variant TGEV, PEDV, and PRCV (388–391).

### Zoonotic Link Between Human and Animal Coronaviruses

In humans, CoVs can cause infections ranging from the common cold to highly pathogenic diseases such as SARS and MERS. The seven human CoVs known to date are HCoV-229E, HCoV-NL63, HCoV-OC43, HCoV-HKU1 causing mild symptoms, and the highly pathogenic MERS-CoV, SARS-CoV, and SARS-CoV-2 causing adverse lower respiratory tract infection. The phylogenetic analysis has shown that bats, mice, or domestic animals serve as gene sources for all the seven HCoV (392). The HCoVs triggering common cold circulate in the human population without any need of an animal reservoir, whereas SARS-CoV and MERS-CoV need to maintain and propagate in their zoonotic intermediate host for possible transmission to human targets. Also, the frequent crossing of species by CoV has led to the emergence of important human pathogens. The key determinants for CoV–host specificity, such as variability in spike glycoprotein, its RBD in different species, and identical nucleotide sequence, provide a framework to understand the switch of hosts and positive selection during inter- and intraspecies transmission events.

The complete genome sequence analysis suggests that BCoV is possibly related to HCoV OC43 (50). It is believed that an interspecies jump was responsible for this genetic similarity, albeit there are no case reports suggesting infection resulting in disease by BCoV in humans. The findings of various studies suggest cross-protection among HCoV and BCoV and evidence of viral RNA in the human nasal mucosa for a short duration after exposure to BCoV (393). A human enteric CoV strain HECV-4408 isolated from a child suffering from acute diarrhea was passaged four times in HRT-18 cells and was inoculated orally in four gnotobiotic calves followed by challenging with BCoV-DB2 strain. The calves inoculated with HECV-4408 developed diarrhea and mild clinical signs along with detection of virus in the feces and nasal shedding by RT-PCR, whereas after BCoV inoculation, no diarrhea or virus shedding was observed in the calves. This experiment fairly suggests that (i) the less severe clinical signs in calves inoculated with HECV-4408 may be due to naturally lower virulence of HECV in calves or that the virulence was altered by a passage in cell culture, as it affects the efficiency of agglutination of RBCs (120, 127), its intestinal replication (128), antigenic composition (394), and associated mutations in the genome (395). (ii) Fecal and serum IgG titers were detected, which either remained the same or increased 2-fold after the challenge. This indicated that the HECV-4408 inoculation developed a protective immune response such that no replication of the virus was observed after challenging with the BCoV-DB2 strain.

There is no risk suspected yet to the public health from IBV. Humans are not considered as a reservoir for replication of IBV. There is no evidence of transmission of virus between humans and from humans to animals. Various mechanical means leading to infections in chicks have been reported. The persons working in commercial poultry farms may be at risk of getting an infection, but the significance is not known (396). IBV has also been detected in wild birds, which may serve as a vector for transmission between free-living and domestic birds (397). Therefore, the wild birds could feature as essential for recombination events that may further lead to the emergence of variants of concerns for humans. The isolation of avian IBV such as viruses from a man with a morphological resemblance with 229E strain of human CoV was reported, which was later confirmed to be an isolate of HCoV and not otherwise (398). This illustrates a likely possibility of the origin of avian and human CoVs from a common gene source resulting in divergent evolution.

The PEDV is genetically closely related to HCoV-229E than to other α-CoVs, and it can also be cultured in Vero cells such as SARS-CoV (399). It is relevant to mention that the swine had always been a predominant species for the evolution of outbreak-causing viruses such as new strains of influenza A virus and are also found to be infected by bat CoVs. The role of pigs and the possibility of the emergence of new strains of viruses, including human CoVs, should not be overlooked and need explorative studies (32, 400, 401). However, Shi et al. (402) had reported that there is no significant susceptibility of pigs to SARS-CoV-2.

The wild mammals and birds harbor CoV resulting in undetected transmission. Genetically similar human CoVs were identified in civets, raccoons, and horseshoe bats (403), and an experimental infection caused disease in macaques, ferrets, and subclinical infection in cats (404, 405). The CoVs in these animals show nucleotide sequence homology (88–90%) with other HCoVs and use ACE2 receptors to bind to spike glycoprotein for entry as seen with SARS-CoV and SARS-CoV-2 (406, 407). These findings suggest their role as intermediate or reservoir hosts. Bats are found to be reservoir hosts for pathogenic HCoV but not as immediate hosts due to few sequence divergence (408). Similarly, the highest genome sequence homology has been found in RBDs of SARS-CoV-2, pangolins, and bats, but still, there is no direct supportive evidence for their origin from a common ancestor, and thus, the evolutionary pathway remains obscure (392). However, a study on analysis of the evolutionary origin of SARS using phylogenetic analysis suggested an avian-like origin for nucleocapsid and matrix protein, mammalian-like origin for replicase proteins, and a mosaic of mammalian–avian-like origin for spike protein. The spike gene was also subjected to a bootscan recombination analysis that revealed high nucleotide sequence similarity between the SARS virus and FIPV (409).

MERS also serves as another example where interspecies transmission is important. The dromedary camels of Middle East countries in Asia and Africa, such as Egypt and Qatar, were detected with MERS-CoV isolated from their nasal swabs and were found seropositive with neutralizing antibodies for MERS-CoV species (410–416). Also, the study on experimental MERS infection in camels revealed massive shedding of a large amount of virus through not only respiratory route but also feco-oral route and milk, suggesting the risk of foodborne transmission to humans and occupational exposure (417–419). This indicates that the camels serve as the *bona fide* reservoir host of MERS-CoV. In a case report, the full genome sequences of two isolates obtained from a dromedary camel with rhinorrhea and a human in close contact with the camel were identical and positive for MERS-CoV RNA (420). The rate of secondary transmission within humans is also observed to be low, only up to 5% (421). A case of a 39-year-old male who developed fever and cough with a history of close contact with dromedary camels at his farm tested positive for MERS-CoV by RT-PCR (422). However, other study reports suggest there is no contact history with camels before the onset of symptoms in many confirmed cases of MERS in humans (423), which attributed to either human-to-human transmission or other transmission routes involving unknown animal species paving the way for further investigations.

The phylogenetic analyses on the molecular level reveal that SARS-CoV-2 is close to SARS-CoV with bat origin and shares 50–51.8% and 79% identity with MERS-CoV and SARS-CoV, respectively (32, 424–426). The recent reports on the detection of SARS-CoV-2 with mild respiratory illness in animals such as cats, dogs, and tigers in contact with humans suggest true infection caused by human-to-animal transmission (427). The two dogs reported from Hong Kong and a dog in New York living in close contact with their SARS-CoV-2 positive owners tested positive through RT-PCR in both nasal and oral samples (428–430). Seroconversion in dogs was observed when the blood samples were tested in the later stages, with weak positive results indicating low viral infection that resulted in the production of antibodies against SARS-CoV-2. Similarly, cats can be found susceptible to SARS-CoV-2 through experimental inoculation and can spread infection through droplets (402, 431). A serological study on the immune response of cats against SARS-CoV-2 revealed high titers of neutralizing antibodies (432). The big feline such as tigers in proximity to asymptomatic positive zookeepers of Bronx Zoo in New York City tested positive for SARS-CoV-2 after showing mild respiratory symptoms (433). Recently, two cases of human-to-cat transmission of SARS-CoV-2 were identified during a screening program of a feline population of households in the United Kingdom (434). The first case of transmission of SARS-CoV-2 from an asymptomatic human carrier, probably caretakers, to eight Asiatic lions at Hyderabad's Nehru Zoological Park was reported in India (435).

The high percent identity between feline and human ACE2 protein sequences, particularly within the receptor-binding interface region, may explain the incidence of cross-species transmission between them. The ferrets were also found to harbor SARS-CoV-2 infection with isolation of virus from the upper respiratory tract and development of mild clinical signs during experimental investigations (402). Therefore, SARS-CoV-2 infection in these companion animals demonstrates their susceptibility under natural and experimental settings. Other farmed animals such as minks, when infected with SARS-CoV-2, may show respiratory and gastrointestinal signs with increased mortality (436, 437). There is evidence that SARS-CoV-2 has evolved at the genetic level into a variant strain in minks in Denmark, and mink-to-mink and mink-to-human transmissions have been observed on farms of Denmark and the Netherlands, whereas ferrets have been reported to transmit the virus to other ferrets in an experimental study (432, 437). Natural SARS-CoV-2 infection in gorillas of San Diego Zoo in California (438) and Asian small-clawed otters (439) have also been reported with high susceptibility to the infection and clinical signs. Experimental investigations on SARS-CoV-2 infection in raccoon dogs, rabbits, pigs, cattle, poultry, hamsters, fruit bats, white-tailed deer, macaques, voles, and mice suggested low to high susceptibility, none to mild clinical signs, and pieces of evidence of low transmissibility between respective animals (440–447). These findings, however, do not hold any substantial evidence for zoonoses, as the transmissibility is low due to insufficient viral load and its replication (427, 431). The viruses in these animals do not satisfy Koch's postulates, and thus, it is inconclusive to declare these species as reservoir hosts for SARS-CoV-2 (448).

### Approaches to Study Interspecies Spillover, Evolution and Zoonoses

The novel animal and human CoVs are the outcomes of recombination, divergence, and subsequent evolution. An efficient genomic, phylogenetic, evolutionary rate, and divergence time analyses are now possible with improved bioinformatics tools and the availability of a large pool of CoVs discovered over time. This is done using different approaches incorporating molecular clock analysis or similar techniques that evaluate mutation rates of biomolecules such as DNA, RNA, or amino acid sequences for proteins. The ecological approaches focus on the spatial distribution of pathogen and host populations and their interactions with each other and their environment. Molecular approaches rely on genetic and cellular aspects of the host–pathogen relationship at the individual and population levels. A combination of these approaches, epidemiological data, and whole-genome sequencing along with some anticipative strategies are essential to clarify the mechanisms by which the virus jumps from one species to another. Interspecies transmission and emergence of novel CoVs are studied through molecular epidemiology, which describes the distribution of genes or their variants by considering parameters such as place, time, and population. Moreover, phylogenomic analysis intersects genomics, origin, and evolution of CoVs carried out exclusively after outbreaks of SARS in humans. This analysis provided evidence for interspecies transmission events such as the emergence of HCoV-OC43 from bovines to humans, TGEV from CCoV-II, FCoV-II, and CCoV-II from recombination between early CCoV-I and FCoV-I and porcine CoV HKU15 likely from a sparrow. Many researchers use the Rob Lanfear's method (449) and the maximum likelihood method with FastTree software (MicrobesOnline, Berkeley, CA, USA) to construct a phylogenetic tree and sequence alignment with the best setting determined by Global Initiative for Sharing All Influenza Data database (https://www.gisaid.org/) and Nextstrain (https://nextstrain.org) (450). Although Bayesian Evolutionary Analysis Sampling Trees is the most widely used approach, it uses different genes (RdRp, ORF1ab, S, N, and helicase genes) and molecular datasets for the purpose to estimate the considerable difference in the life history and evolution of CoVs (1, 22, 451). Klompus et al. demonstrated interspecies cross-reactivity mediated by reactive monoclonal antibodies that bind to antigens from human CoVs (hCoV-OC43 and hCoV-HKU1) and several animal CoVs with shared motifs to SARS-CoV-2. This serological strategy based on cross-reactive antibody responses along with DNA sequencing and RT-qPCR testing has been a dominant methodology and potential diagnostic strategy for infections of novel CoV spillovers (452).

## Conclusion

CoV has a wide host range affecting domestic livestock, wild animals, and birds, often resulting in serious disease outcomes and economic losses. Furthermore, pets and various livestock species share a common environment with humans and continue to pose a threat to the public at risk. The presence of antibodies against animal CoVs in humans could merely be a normal immune response to an occasional or occupational exposure without any apparent disease. However, frequent and prolonged exposure to animal CoVs and the need for the virus to mutate to bypass the protective host-immune responses would likely result in newer CoV strains with a better ability to infect immunocompromised human hosts. Interestingly, it has been observed that companion animals are susceptible hosts for human CoVs such as SARS-CoV-2. The similarities in the CoV key proteins having a role in initiating infections, together with genetic and evolutionary relatedness and habitat sharing by diverse hosts, will drive future disease outcomes and virus evolution. At present, there is no strong evidence suggesting the role of livestock, poultry, and pets' CoVs to cause serious disease in humans; however, ample scope to undergo mutations and cross-jump species barrier to cause life-threatening illnesses exists. The enigmatic nature of CoVs to cause alteration in tissue tropism, jump species barriers, and form variants is remarkable and needs elucidation. CoVs tend to rapidly adapt to changing ecological niches and enhance the possibility of the emergence of novel CoVs due to high mutational errors particularly in the RBD of spike protein, along with inconstancy of replication enzymes and cleavage sites. These errors in S protein, important for host receptor usage, are essential for the emergence of mutants and the establishment of an effective and productive human infection. Consequently, the occurrence of double and triple mutants has made researchers focus on establishing epidemiological linkage to correlate these variants of concerns with the existing public health scenario. Furthermore, the proven propensity of CoVs for interspecies transmission, to emerge potentially from unknown reservoirs and to genetically relate with CoVs from different hosts, indicates the continued introduction of animal CoVs into the human population. The outbreaks of SARS-CoV in the year 2002 and MERS-CoV in 2012 in the form of severe acute respiratory distress had less impact than the current pandemic caused by SARS-CoV-2 that accounted for nearly 216.30 million confirmed cases with a death toll nearing four million by August 30, 2021, globally (453). Although these numbers are way higher, the case fatality rate of SARS-CoV-2 is around 4%, which is still lower than SARS-CoV and MERS-CoV outbreaks, which ranged between 0 and 50% (454–456). The genome of novel SARS-COV-2 is chimeric in the sense that the majority of it shares homology with the bat CoV genome, whereas the RBD portion bears a sequence similar to pangolin CoV. It is this specific RBD sequence that enables SARS-COV-2 to bind with high affinity to hACE2, resulting in productive infection and deaths. This is an unambiguous example of how recombination and mutation events result in highly virulent strains claiming lives and damaging economies.

The definitive understanding of the origin of SARS-CoV-2 will have marked repercussions on how humans interact with the ecosystem and on laboratory practice policies and biosafety regulations. Therefore, the emergence and evolution of novel CoVs because of their widespread association and frequent infections in animal hosts, both livestock and wild, combined with a high mutation rate, need to be contemplated to meet future challenges. There is an impending need to implement an effective strategy and monitor animal coronavirome as a potential reservoir of CoV reintroduction to humans. Large-scale sampling, metagenomic sequencing, and comparative genome analysis from animals of different geographical locations, especially those in frequent contact with human and wildlife habitats, should be considered. Baseline samples of unexposed individuals, preferably from a pre-pandemic population, need to be collected to compare with the individuals infected with new spillovers. The establishment of phage antibody libraries for profiling antibody responses against novel CoVs and enhancing the immediate availability of animal CoV serological assays will assist in the screening of numerous antigens in a critically early phase of future outbreaks (452). Despite the availability of established and novel diagnostic technologies, the detection of animal CoVs is still based on conventional, time-consuming, and less-sensitive molecular techniques. Therefore, there is a need to drive efforts for the development of rapid, cost-effective, and sensitive laboratory or POC diagnostic tools with a multi-prong approach to potentially increase the efficiency and specificity of diagnosis. These upgraded testing efforts will help in limiting the spread of animal CoV infections in farms, multi-pet households, and wildlife niches. The commonly used whole cell-based vaccine strategies to combat animal CoV infections on the field deliver poor protection in farm and pet animals. This calls for the exploitation of viral-vectored and nucleic acid-based vaccines as promising candidates. The knowledge about factors responsible for the success or failure of animal CoV vaccines can also provide better insight into the design of vaccines for humans against pandemic-causing CoVs. The standard measures used for the prevention of infectious diseases at farms and elsewhere should be followed besides maintaining personal hygiene and avoiding contact with wildlife to restrict exposure to animal CoVs. Needless to mention that the development of novel diagnostics, vaccines, detailed epidemiological studies, and sequencing of variants of interest and concern will, of course, need budgetary provisions.

## Author Contributions

PP: reviewed the literature and wrote the manuscript. SV: finalized the manuscript. All authors contributed to the article and approved the submitted version.

## Conflict of Interest

The authors declare that the research was conducted in the absence of any commercial or financial relationships that could be construed as a potential conflict of interest.

## Publisher's Note

All claims expressed in this article are solely those of the authors and do not necessarily represent those of their affiliated organizations, or those of the publisher, the editors and the reviewers. Any product that may be evaluated in this article, or claim that may be made by its manufacturer, is not guaranteed or endorsed by the publisher.
